# Vitamin D deficiency is associated with IL-6 levels and monocyte activation in HIV-infected persons

**DOI:** 10.1371/journal.pone.0175517

**Published:** 2017-05-02

**Authors:** Maura Manion, Katherine Huppler Hullsiek, Eleanor M. P. Wilson, Frank Rhame, Erna Kojic, David Gibson, John Hammer, Pragna Patel, John T. Brooks, Jason V. Baker, Irini Sereti, for the Study to Understand the Natural History of HIV/AIDS in the Era of Effective Antiretroviral Therapy (the ‘SUN Study’) Investigators

**Affiliations:** 1 National Institute of Allergy and Infectious Disease, National Institutes of Health, Bethesda, Maryland, United States of America; 2 Division of Biostatistics, University of Minnesota, Minneapolis, Minnesota, United States of America; 3 Institute of Virology, University of Maryland School of Medicine, Baltimore, Maryland, United States of America; 4 Department of Medicine, University of Minnesota, Minneapolis, Minnesota, United States of America; 5 Abbott Northwestern Hospital, Minneapolis, Minnesota, United States of America; 6 Miriam Hospital, Providence, Rhode Island, United States of America; 7 Washington University, St. Louis, Missouri, United States of America; 8 Denver Infectious Disease Consultants, Denver, Colorado, United States of America; 9 Division of HIV/AIDS Prevention, Centers for Disease Control and Prevention, Atlanta, Georgia, United States of America; 10 Division of Infectious Diseases, Hennepin County Medical Center, Minneapolis, Minnesota, United States of America; Emory University School of Medicine, UNITED STATES

## Abstract

**Background:**

Immune activation plays a key role in HIV pathogenesis. Markers of inflammation have been associated with vitamin D deficiency in the general population. Studies have also demonstrated associations of vitamin D deficiency with increased risk of HIV progression and death. The relationship between persistent inflammation and immune activation during chronic HIV infection and vitamin D deficiency remains unclear.

**Methods:**

Cryopreserved specimens were analyzed from 663 participants at the time of enrollment from the Study to Understand the Natural History of HIV/AIDS in the Era of Effective Therapy (SUN Study) from 2004 to 2006. Biomarkers of inflammation, atherosclerosis, and coagulation were measured using enzyme-linked immunosorbent assays (ELISAs) and electrochemiluminescence. 25(OH)D, the stable precursor form of vitamin D, was measured using a radioimmunoassay with levels defined as: normal (≥30ng/mL), insufficient (20–29 ng/mL) and deficient (<20 ng/mL). Monocyte phenotypes were assessed by flow cytometry. Linear and logistic regression models were used to determine statistical associations between biomarkers and vitamin D deficiency.

**Results:**

25(OH)D levels were deficient in 251 (38%) participants, insufficient in 222 (34%), and normal in 190 (29%). Patients with vitamin D deficiency, when compared to those with insufficient or normal vitamin D levels, had increased levels of IL-6 (23%; p<0.01), TNF-α (21%, p = 0.03), D-dimer (24%, p = 0.01), higher proportions of CD14^dim^CD16+ (22%, p<0.01) and CX3CR1+ monocytes (48%; p<0.001) and decreased frequency of CCR2+ monocytes (-3.4%, p<0.001). In fully adjusted models, vitamin D associations with abnormal biomarker levels persisted for IL-6 levels and CX3CR1+ and CCR2+ phenotypes.

**Conclusions:**

Vitamin D deficiency is associated with greater inflammation and activated monocyte phenotypes. The role of vitamin D deficiency in persistent immune activation and associated complications during chronic HIV disease should be further evaluated as a possible target for intervention.

## Introduction

Studies continue to elucidate the role of vitamin D, both its prohormone and its more stable circulating precursor, 25-hydroxyvitamin D (25(OH)D), in inflammatory diseases. Recent data have shown that severe vitamin D deficiency is associated with an increased risk of human immunodeficiency virus (HIV) disease progression and death[1], as well as elevated levels of interleukin-6 (IL-6)[2, 3], a biomarker of innate immune activation. IL-6 elevations have previously been shown to predict mortality and risk for non-AIDS-defining complications among persons with chronic HIV infection[4, 5].

Previous work by our group investigating inflammation in HIV-infected adults has shown biomarkers of innate immune activation correlate strongly with monocyte activation phenotypes and only modestly with phenotypes of T-cell maturation, but not with markers of CD8 T-cell activation[6]. In addition, monocyte activation was independently associated with progression of coronary artery calcifications after adjusting for traditional cardiac risk factors[7]. In the present analysis, our aim was to evaluate the associations of vitamin D deficiency with cellular phenotypes of adaptive and innate immune activation to further explore the mechanistic pathways linking vitamin D with innate immunity and the chronic inflammation that persists in treated HIV infection.

## Methods

### Study design

The Study to Understand the Natural History of HIV/AIDS in the Era of Effective Antiretroviral Therapy (the SUN Study) was a Centers for Disease Control and Prevention (CDC)-funded prospective observational cohort study of HIV-infected participants enrolled in four United States (U.S.) cities (Denver, Minneapolis, Providence, and St. Louis) between March 2004 and August 2006[8]. The protocol was approved by the institutional review boards of the CDC (the CDC IRB-B) and each participating institution(Hennepin County Medical Center, Park Nicollet Hospitals and Clinics, Abbott Northwestern Hospital, Miriam Hospital, Washington University, and Denver Infectious Disease Consultants). All participants provided written informed consent. (NCT #00146419) Eligible participants were expected to survive at least two years and were either treatment naïve or exposed solely to combination ART (≥ 3 nucleoside reverse transcriptase inhibitors [NRTI] or ≥ 3 antiretroviral drugs from at least 2 different classes).

Clinical data, including all medications and diagnoses, were extracted from medical charts and entered into a single database (Clinical Practice Analyst; Cerner Corporation, Vienna, VA). Additional data were collected through study-specific physical examinations, laboratory testing, and an audio computer-assisted self-interview[8].

### Specimen collection and laboratory measurements

At study entry, fasting whole blood and plasma specimens were collected and shipped overnight to the CDC. Peripheral blood mononuclear cells (PBMC) were isolated and cryopreserved centrally at a CDC lab within 30 hours of blood draw. All specimens were then stored in liquid nitrogen at -70°C until analyzed. Clinical site laboratory testing included measurement of fasting serum lipids, plasma HIV RNA viral load (VL), and CD4 cell counts.

### Measurement of biomarkers in cryopreserved plasma

All biomarkers were measured on the study entry specimens. Vitamin D was measured as 25(OH)D with the 25(OH)D iodine 125 radioimmunoassay (Diasorin, Saluggia, Italy). The following soluble biomarkers were measured at the Diabetes Research and Training Center Radioimmunoassay Core Laboratory (Washington University School of Medicine): IL-8 (BD Biosciences, San Jose, CA) using an ezyme-linked immunosorbent assay (ELISA) based assay, as well as hsCRP (Kamiya Biomedical Company, Seattle, WA) and D-dimer (Roche Diagnostics, Indianapolis, IN) using immunoturbidometric assays on a Hitachi 917 analyzer. We measured sCD14 using an ELISA based assay (R&D Systems, Minneapolis MN). Tumor necrosis factor (TNF)-α was measured using chemiluminescence on the Immulite 1000 (Erlangen, Germany). The following biomarkers were measured at the National Institutes of Allergy and Infectious Diseases: IL-6 (Meso Scale Discovery, Rockville, MD) using electrochemiluminescence immunoassays and sCD163 (Aviscera Bioscience, Inc., Santa Clara, CA) using an ELISA based assay.

### Immunophenotyping of PBMC

Immunophenotyping was performed on cryopreserved PBMC on the study entry specimens using multi-color flow cytometry as previously described[6]. The fluorochrome-conjugated antibodies used to evaluate monocyte phenotypes were: anti-CD2 efluor450 (Clone:RPA-2.10), anti-CD3 efluor450 (clone:UCHT1), anti-CD19 efluor450 (Clone:HIB19), anti-CD20 APC (Clone:2H7), and anti-HLADR efluor605 (Clone:LN3) from eBioscience; anti-CX3CR1 APC (Clone:2A9-1), anti-CD16 PE-Cy7 (Clone:3G8), and anti-CCR2 PerCp-Cy5.5 (Clone:TG5) from BioLegend; anti-CD14 PE (Clone:M5E2) and anti-CCR5 APC-Cy7 (Clone:2D7) from BD Biosciences, anti-TF FITC (Clone:VIC7) from American Diagnostica; and Live/Dead Fixable Blue Dead Cell Stain Kit with Ultra Violet (UV) excitation from Invitrogen.

The fluorochrome-conjugated antibodies used to evaluate T-cell phenotypes: anti-CD38 PE-Cy7 (Clone:HIT2), anti-CX3CR1 PE (Clone:2A9-1), anti-CD28 PE-Cy7 (Clone:CD28.2), and anti-CD4 efluor605 (Clone:OKT4) from eBioscience, anti-CD57 APC (Clone:HCD57) and anti-CD27 Ax700 (Clone:O323) from BioLegend, anti-HLADR PE (Clone:G46-6), anti-CD3 APC-Cy7 (Clone:SK7), anti-CD8 PB (Clone:RPA-T8), and anti-CD56 PB (Clone:B159) from BD Biosciences, and anti-CD45RO ECD (Clone:UCHL1) from Beckman Coulter.

Samples were acquired on an LSR-II flow cytometer (Becton Dickinson, Franklin Lakes, NJ) and data were analyzed using FlowJo software version 9.3.3 (Treestar Inc., Ashland, OR). The proportion of monocytes are expressed as a percent, with the following cell types characterized: classical monocyte phenotype (CD14^++^CD16^-^), intermediate monocyte subset (CD14^+^CD16^+^), or patrolling monocytes (CD14^dim^CD16^+^), monocytes expressing tissue factor (TF), and monocytes expressing markers of tissue migration (CCR2^+^ and CX3CR1+). Gates were established as previously described [6]. All samples had >75% viability; only live cells were included in all analyses.

### Statistical methods

Participants’ vitamin D levels at study entry were categorized as normal (≥30ng/mL), insufficient (20–29 ng/mL) or deficient (<20 ng/mL) per the United States Endocrine Society[9]. Study entry characteristics were summarized with frequencies or medians and interquartile ranges (IQR), as appropriate. Demographic data were compared among the three vitamin D groups with the chi-square and Kruskal-Wallis tests. For each biomarker and monocyte population the overall strength of association with concentration of vitamin D was determined with Spearman's correlation. The biomarkers were normalized for analysis with a log_2_ transformation. The relative percent difference between those with deficient versus non-deficient vitamin D levels was assessed with general linear models. Finally, univariate and multivariable logisitic regression models were used to explore the associations with biomarkers and vitamin D deficiency (<20ng/mL). Odds ratios expressing the risk of vitamin D deficiency per doubling of the biomarker were calculated with their corresponding 95% confidence intervals. Multivariable models were adjusted for baseline covariates that differed between vitamin D groups: age, gender, race, Body Mass Index (BMI), treatment for hypertension (HTN), non-nucleoside reverse transcriptase inhibitor (NNRTI) regimen, CD4 cell count, and time since HIV diagnosis.

## Results

### Participant characteristics

Sufficient study entry plasma and PBMCs were available for 663 out of 700 participants. Vitamin D levels were deficient for 251 (38%) participants, insufficient for 222 (34%), and normal for 190 (29%) (Table 1). As has been previously reported in Dao et al [10], SUN Study participants differed in terms of age, gender, race, BMI, treatment for HTN, current CD4 cell count, and time since HIV diagnosis across the three vitamin D groups (Table 1). Other characteristics were similar across the groups, including smoking status, intravenous drug use as an HIV transmission risk factor, and the prevalence of diabetes, hyperlipidemia, and chronic hepatitis. Clinical characteristics also did not differ by vitamin D status, including nadir CD4 T-cell count, a prior AIDS diagnosis, and HIV VL < 400 copies/mL that were similar across the cohort regardless of vitamin D level. There were significantly more patients with vitamin D insufficiency and deficiency who were prescribed an NNRTI-based regimen, whereas more Vitamin D replete patients were prescribed Protease Inhibitor (PI) based regimens. Among participants prescribed a NNRTI, 68% were prescribed efavirenz whereas among participants prescribed a PI, 42% were prescribed atazanavir and 40% lopinavir.

**Table 1:** Participant characteristics, the Study to Understand the Natural History of HIV/AIDS in the Era of Effective Therapy, United States, 2004–2006 (n = 663).

	Vitamin D Level at Enrollment			
	Normal	Insufficient	Deficient	P-value
	(≥ 30ng/mL)	(20–29 ng/mL)	(< 20 ng/mL)	
No. People	190 (28.7%)	222 (33.5%)	251 (37.9%)	
(% of Total Participants)				
	n (%)	n (%)	n (%)	
Male Gender	158 (83.2)	176 (79.3)	170 (67.7)	<0.001
Race				<0.001
• White	148 (77.9)	140 (63.1)	95 (37.8)	
• Black	26 (13.7)	54 (24.3)	121 (48.2)	
• Other	16 (8.4)	28 (12.6)	35 (13.9)	
Diabetic	12 (6.3)	20 (9.0)	27 (10.8)	0.27
Hypertensive	46 (24.2)	76 (34.2)	88 (35.1)	0.03
On treatment for HTN	23 (12.1)	49 (22.1)	54 (21.5)	0.02
On treatment for HDL	16 (8.4)	22 (9.9)	17 (6.8)	0.47
Prior AIDS	52 (27.4)	49 (22.1)	62 (24.7)	0.46
Current smoker*	87 (46.5)	90 (41.7)	108 (44.4)	0.61
Injection drug* use	24 (12.8)	34 (15.7)	30 (12.3)	0.53
On ART^1^	153 (80.5)	180 (81.1)	190 (75.7)	0.43
• NNRTI based^2^	57 (37.3)	89 (49.4)	109 (57.4)	0.001
• PI based^2^	93 (60.8)	78 (43.3)	67 (35.3)	<0.0001
• Abacavir-containing^2^	61 (39.9)	66 (36.7)	62 (32.6)	0.19
• Tenofovir-containing^2^	99 (64.7)	95 (52.8)	88 (46.3)	0.001
HIV RNA (< 400 c/mL^2^)*	139 (90.8)	158 (88.3)	166 (87.8)	0.65
Not on ART	37 (19.5)	42 (18.9)	61 (24.3)	
• Prior Use^3^	9 (24.3)	21 (50.0)	33 (54.1)	0.02
• No prior use^3^	28 (75.7)	21 (50.0)	28 (45.9)	
	Median [IQR]	Median [IQR]	Median [IQR]	
Age (years)	41 [36, 46]	42 [36, 49]	40 [33, 46]	0.02
Body mass index (kg/m^2^)	25 [23, 27]	26 [23, 29]	27 [23, 31]	<0.01
Nadir CD4 cell count	192 [60, 311]	205 [107, 310]	216 [90, 333]	0.37
(cells/mm3)				
Current CD4 cell count	431 [322, 619]	498 [349, 729]	473 [331, 693]	0.01
(cells/mm3)				
Duration on ART (months)				
Current ART	26 [12, 59]	33 [15, 70]	41 [15, 65]	0.08
Prior ART	9 [3, 42]	34 [12, 44]	19 [8, 47]	0.47
Time since HIV diagnosis	46 [19, 93]	62 [28, 95]	62 [32, 101]	0.01
(months)				

Values reported as N (%), or Median [IQR = interquartile range]

Chi-squared test of proportions or Kruskall Wallis non-parametric tests

^1^ Rows are not mutually exclusive

^2^ Among patients on ART

^3^ Among participants not on ART

*Missing variable data with new denominator:

Current smoker: Normal = 187, Insufficient = 216, Deficient = 243

Injection Drug Use: Normal = 187, Insufficient = 216, Deficient = 243

HIV VL <400: Insufficient = 179, Deficient = 189

HTN = Hypertension, HDL = High-density lipoprotein, ART = Antiretroviral therapy, NNRTI = Nonnucleoside reverse transcriptase inhibitor, PI = Protease Inhibitor

### Plasma biomarkers

**Fig 1:**
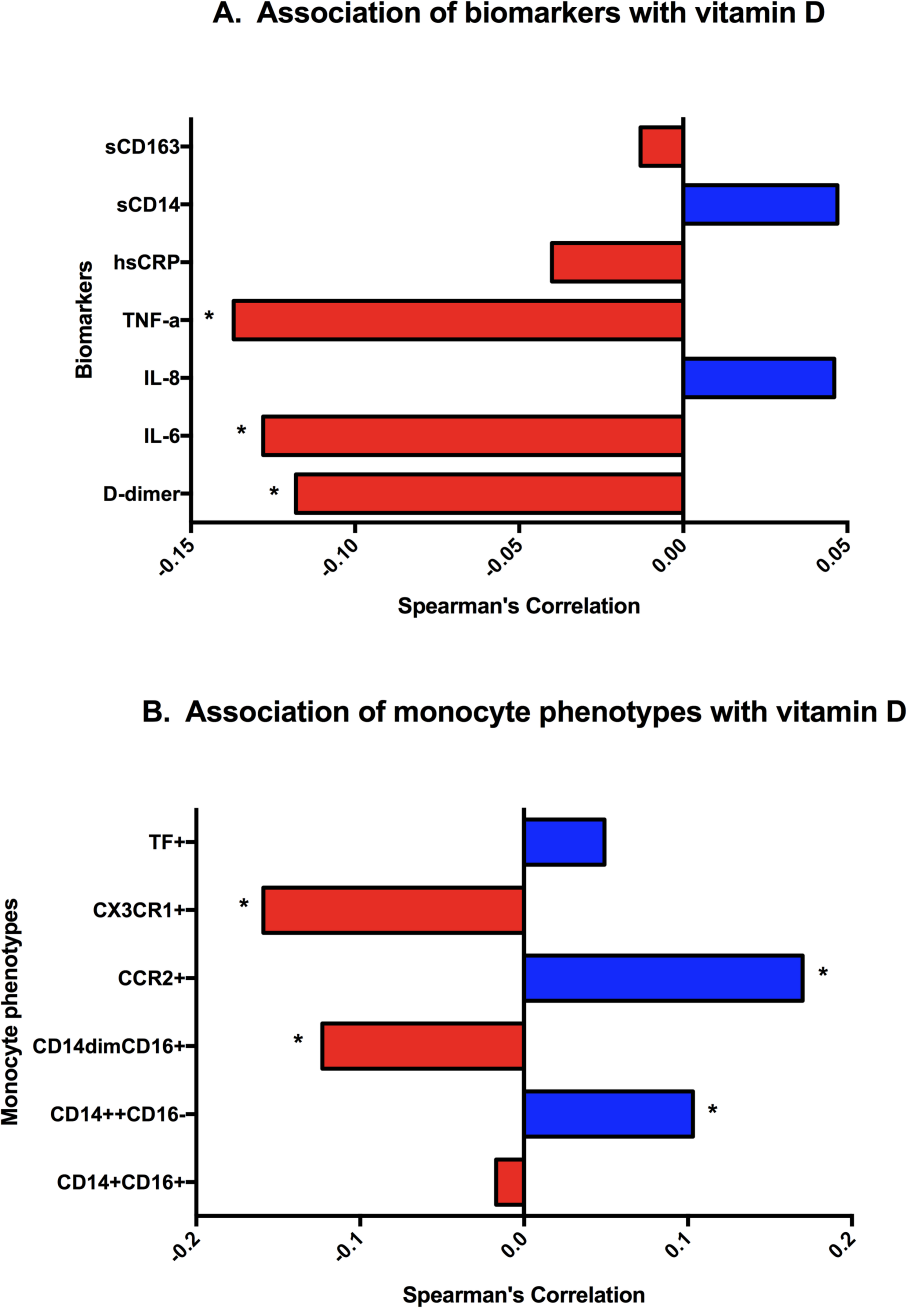
Baseline spearman correlations with vitamin D levels. A) Association of biomarkers with vitamin D (Vitamin D levels are based upon the measured levels per patient, *p<0.01)) B) Association of monocyte phenotypes with vitamin D (Vitamin D levels are based upon the measured levels per patient, * p<0.01).

Vitamin D was significantly inversely correlated with plasma levels of D-dimer, IL-6, and TNF-α. (p <0.001 for each, Fig 1A and S1A Fig). Vitamin D-deficient participants compared with patients who had normal and insufficient levels had increased levels of D-dimer (24%; p = 0.04), IL-6 (23%; p<0.01), and TNF-α (21%; p = 0.04) (Table 2). Levels of other plasma biomarkers did not differ significantly between those with deficient versus non-deficient vitamin D status.

**Table 2:** Geometric means (with 95% CI) of plasma biomarkers, monocytes and T-cell phenotypes, by vitamin D levels and percent difference of deficient to those not deficient, at study enrollment, the Study to Understand the Natural History of HIV/AIDS in the Era of Effective Therapy, United States, 2004–2006

Vitamin D Level at Enrollment					
	Normal	Insufficient	Deficient	% Difference^1^	P-value^2^
	(≥ 30 ng/mL)	(20–29 ng/mL)	(< 20 ng/mL)		
Plasma Biomarkers					
D-Dimer (mg/L)	0.2 [0.2, 0.2]	0.2[0.2, 0.3]	0.3 [0.2, 0.3]	19.1 [0.6, 41.1]	0.04
IL-6 (pg/mL)	1.2 [1.1, 1.4]	1.3[1.2, 1.5]	1.5 [1.4, 1.7]	23.3 [6.4, 42.9]	<0.01
IL-8 (pg/mL)	16.2 [14.2, 18.5]	15.5 [13.9, 17.4]	15.2 [13.7, 16.9]	-4.0 [-16.3, 10.2]	0.56
TNF-α (pg/mL)	17.3 [14.9, 20.1]	19.1 [16.6, 21.9]	21.7 [18.9, 24.8]	18.8 [0.5, 40.6]	0.04
hsCRP (mg/L)	2.0 [1.7, 2.3]	2.2 [1.9, 2.5]	2.3 [2.0, 2.6]	8.0 [-10.3, 30.1]	0.42
sCD14 (ng/mL)	1169.1 [1127.6, 1212.1]	1127.3 [1089.6, 1166.3]	1149.3 [1106.7, 1193.6]	0.3 [-4.0, 4.7]	0.91
sCD163 (pg/mL)	542.1 [498.3, 589.8]	578.0 [522.2, 639.8]	539.6 [481.6, 604.5]	-4.1 [-15.4, 8.7]	0.51
Monocyte phenotypes					
(% of Monocytes)					
% CD14++CD16+	5.1 [4.6, 5.6]	4.9 [4.4, 5.4]	5.2 [4.8, 5.7]	3.3 [-7.1, 14.9]	0.55
% CD14+CD16-	77.3 [75.5, 79.1]	75.8 [73.0, 78.7]	75.3 [73.9, 76.8]	-1.5 [-4.7, 1.8]	0.36
% CD14^dim^CD16+	5.4 [4.9, 6.1]	5.6 [5.0, 6.3]	6.7 [6.1, 7.4]	20.0 [5.4, 36.7]	<0.01
% CCR2+	88.5 [87.5, 89.6]	88.6 [87.6, 89.6]	85.6 [84.4, 86.8]	-3.4 [-4.8, -1.9]	<0.001
% CX3CR1+	5.3 [4.5, 6.1]	5.4 [4.7, 6.2]	7.4 [6.6, 8.3]	45.1 [24.7, 68.8]	<0.001
% TF+	1.9 [1.7, 2.2]	1.9 [1.6, 2.2]	1.7 [1.5, 1.9]	-12.7 [-26.3, 3.4]	0.12
T-cell phenotypes					
(% of CD4 and CD8)					
% CD4+CD38+DR+	8.2 [7.3, 9.2]	8.4 [7.6, 9.3]	8.2 [7.4, 9.0]	-2.1 [-13.6, 10.9]	0.74
% CD8+CD38+DR+	20.0 [17.8, 22.4]	18.7 [17.1, 20.4]	19.9 [18.1,21.9]	1.9 [-9.1, 14.2]	0.75
% CD4+CD28-CD57-	2.0 [1.7, 2.3]	1.9 [1.6, 2.3]	1.7 [1.5, 2.0]	-10.9[-28.3,10.6]	0.30
% CD8+CD20-CD57-	27.5 [25.5, 29.7]	26.1 [24.5, 27.9]	26.0 [24.2, 28.0]	-2.7 [-10.7, 6.0]	0.53

Data are mean and 95% Confidence Interval.

^1^Percent difference comparing Vitamin D deficient to those not deficient.

^2^P-values are from general linear models

In univariate logistic regression models, higher levels of D-dimer, IL-6, and TNF-α were also associated with vitamin D deficiency (Table 3). After adjusting for known baseline differences among the vitamin D groups, only increased IL-6 was associated (p = 0.02) with vitamin D deficiency, and for each doubling of IL-6 there is a 19% increase in the odds of being vitamin D deficient.

**Table 3:** Association of biomarkers and immune phenotypes with vitamin D deficiency, the Study to Understand the Natural History of HIV/AIDS in the Era of Effective Therapy, United States, 2004–2006

	Unadjusted Odds Ratio		Adjusted Odds Ratio	
	Odds Ratio (95%CI)	P-value	Odds Ratio (95%CI)	P-value*
Plasma Biomarkers				
D-dimer (mg/L)	1.25 (1.05, 1.49)	0.01	1.14 (0.94, 1.38)	0.19
IL-6 (pg/mL)	1.18 (1.04, 1.35)	0.01	1.19 (1.03, 1.39)	0.02
IL-8 (pg/mL)	0.96 (0.83, 1.10)	0.56	0.96 (0.83, 1.12)	0.61
TNF-α (pg/mL)	1.11 (1.00, 1.24)	0.05	1.03 (0.92, 1.15)	0.63
hsCRP (mg/L)	1.04 (0.95, 1.15)	0.40	0.94 (0.84, 1.06)	0.31
sCD14 (ng/mL)	1.02 (0.69, 1.52)	0.91	1.24 (0.79, 1.95)	0.35
sCD163 (pg/mL)	0.95 (0.81, 1.11)	0.53	0.88 (0.73, 1.05)	0.16
Monocyte Phenotypes				
% CD14++CD16+	1.06 (0.91, 1.24)	0.45	1.02 (0.86, 1.21)	0.81
% CD14+CD16-	0.79 (0.47, 1.32)	0.36	1.04 (0.60, 1.81)	0.89
% CD14^dim^CD16+	1.23 (1.07, 1.41)	<0.01	1.06 (0.91, 1.24)	0.44
% CCR2+	0.07 (0.02, 0.24)	<0.001	0.16 (0.04, 0.62)	<0.01
% CX3CR1+	1.29 (1.14, 1.47)	<0.001	1.19 (1.04, 1.36)	<0.01
% TF+	0.92 (0.82, 1.02)	0.11	0.91 (0.81, 1.03)	0.13
T-cell Phenotypes				
% CD4 CD38+DR+	0.99 (0.86, 1.13)	0.88	1.02 (0.87, 1.18)	0.83
% CD8 CD38+DR+	1.04 (0.90, 1.20)	0.60	1.05 (0.89, 1.24)	0.56
% CD4 CD28-CD57-	0.94 (0.86, 1.03)	0.21	0.97 (0.88, 1.07)	0.56
% CD8 CD28-CD57-	0.94 (0.77, 1.15)	0.54	1.02 (0.81, 1.28)	0.89

Deficient Vitamin D is value <20

*Adjusted for age, male gender, white race, BMI, treatment for HTN, NNRTI regimen, CD4 cell count, and time since HIV diagnosis

CI = Confidence Interval, BMI = Body Mass Index, HTN = Hypertension, NNRTI = Nonnucleoside reverse transcriptase inhibitor

### Monocyte phenotypes

Vitamin D concentrations were significantly inversely correlated with the percentage of patrolling monocytes (CD14^dim^CD16^+^) and specifically monocytes expressing the patrolling monocyte-associated chemokine, CX3CR1+. Vitamin D concentrations were significantly correlated with the percentage of classical monocytes (CD14^++^CD16^-^) and specifically monocytes expressing the classical-monocyte associated chemokine, CCR2+ (Fig 1B and S1B Fig). Vitamin D-deficient participants compared with those with normal and insufficient levels, had an increased proportion of CD14^dim^CD16+ monocytes (22%; p<0.01), increased proportion of CX3CR1+ monocytes (48%; p <0.001), but a lower proportion of CCR2+ monocytes (-3.4%; p< 0.001; Table 2).

In univariate logistic regression models, we found that increased expression of CX3CR1+ (p <0.01) and decreased expression of CCR2+ (p < 0.01) were significantly associated with vitamin D deficiency (Table 3). In fully adjusted models associations with vitamin D deficiency persisted for CCR2+ and CX3CR1+.

### T-cell phenotypes

T-cell subsets were analyzed for markers of immune activation and senescence. There were no significant associations between these markers and vitamin D levels. (Table 2 and Table 3).

## Discussion

In this study, we explored associations between vitamin D status and levels of soluble and cellular biomarkers of inflammation that predict clinical risk of mortality and non-AIDS-defining complications among participants in a contemporary cohort of HIV-infected persons at low risk for AIDS. As our understanding of the complex role vitamin D plays in regulating innate and adaptive immune responses evolves, identifying the cellular targets of the active form of vitamin D and the relationship with inflammatory biomarkers is an important step. Recent studies have implicated 25(OH)D deficiency in HIV disease progression and death[1], and further work has associated the phenotype of vitamin D deficiency with soluble markers of inflammation that predict mortality [2, 3].

Here, we evaluated cellular phenotypes that may link vitamin D and biomarkers of inflammation with clinical endpoints in chronic treated HIV infection. In univariate analysis, we demonstrated an association of D-dimer, IL-6 and TNF-α with vitamin D deficiency, suggesting a relationship between vitamin D and inflammation.

Among patients with vitamin D deficiency, we also found higher percentages of patrolling monocytes (CD14^dim^CD16+), which typically express high levels of CX3CR1, and lower percentages of the classical monocyte subset (CD14++CD16-) that typically express CCR2 overall. After adjustment, greater expression of CX3CR1 and lower expression of CCR2 remained significantly associated with vitamin D deficiency. The finding of diminished CCR2 expression was consistent with prior findings suggesting a downregulation of CCR2 with systemic inflammation[6, 11]. The increase of CX3CR1 on monocytes has also been associated with systemic inflammation in HIV [11, 12]. These shifts in monocyte phenotypes demonstrate a potential link between vitamin D deficiency and immune activation.

In contrast to other reports, we did not observe an increase in activated T-cell phenotypes associated with vitamin D deficiency [13], however, our participants included Americans of different ethnicities, without excluding skin phototypes, which could play a role in the differences in T-cell phenotypes we observed.

Vitamin D deficiency is prevalent in both the general and HIV-infected populations[10], and we report a prevalence of more than 35% in the present analysis. 25(OH)D deficiency has been reported to increase susceptibility to tuberculosis and other infectious diseases [14], as well as a variety of chronic diseases, including bone disease, muscle weakness, diabetes, cardiovascular disease, and some malignancies in HIV-uninfected persons [15, 16]. However, the implications for persons with chronic HIV infection who are at relatively low risk for tuberculosis or other opportunistic infections have not been as well studied. Active forms of vitamin D and its prohormone can regulate maturation of innate immune cells, including monocytes and dendritic cells [17]. The extent to which vitamin D supplementation may affect monocyte function, cytokine production, or chronic inflammation in the absence of acute and uncontrolled mycobacterial infection is unknown [17].

Our study was limited by the lack of an HIV-uninfected control group, which would be useful to determine whether these immunologic associations differ by HIV infection status. We could infer neither causation nor control for unmeasured confounding because of the cross-sectional and non-randomized study design. The SUN Study also did not have sufficient numbers of participants or follow-up time to study associations directly with rates of clinical events.

Vitamin D insufficiency and deficiency were prevalent in this U.S. HIV cohort with high CD4 cell counts and high levels of viral suppression. Deficient levels of vitamin D were independently associated with activated monocyte phenotypes. The role of vitamin D deficiency in persistent immune activation and associated complications during chronic HIV disease should be further evaluated and considered as a possible target for intervention.

## Supporting information

S1 Fig Scatterplots of biomarkers and monocyte phenotypes. S1 Fig A demonstrates scatter plots of biomarkers by vitamin D and S1 Fig B demonstrates scatter plots of monocyte phenotypes by vitamin D.
(TIFF)

## References

[1] Havers F, Smeaton L, Gupte N, Detrick B, Bollinger RC, Hakim J, et al 25-Hydroxyvitamin D insufficiency and deficiency is associated with HIV disease progression and virological failure post-antiretroviral therapy initiation in diverse multinational settings. The Journal of infectious diseases. 2014;210(2):244–53. PubMed Central PMCID: PMCPMC4141201. doi: 10.1093/infdis/jiu259 24799602 10.1093/infdis/jiu259 PMC4141201

[2] Shepherd L, Souberbielle JC, Bastard JP, Fellahi S, Capeau J, Reekie J, et al Prognostic value of vitamin D level for all-cause mortality, and association with inflammatory markers, in HIV-infected persons. The Journal of infectious diseases. 2014;210(2):234–43. doi: 10.1093/infdis/jiu074 24493824 10.1093/infdis/jiu074

[3] Ansemant T, Mahy S, Piroth C, Ornetti P, Ewing S, Guilland JC, et al Severe hypovitaminosis D correlates with increased inflammatory markers in HIV infected patients. BMC infectious diseases. 2013;13:7 PubMed Central PMCID: PMC3545895. doi: 10.1186/1471-2334-13-7 23295013 10.1186/1471-2334-13-7 PMC3545895

[4] Kuller LH, Tracy R, Belloso W, De Wit S, Drummond F, Lane HC, et al Inflammatory and coagulation biomarkers and mortality in patients with HIV infection. PLoS medicine. 2008;5(10):e203 PubMed Central PMCID: PMC2570418. doi: 10.1371/journal.pmed.0050203 18942885 10.1371/journal.pmed.0050203 PMC2570418

[5] Grund B, Baker J, Deeks SG, Wolfson J, Wentworth D, Cozzi-Lepri A, et al., editor Combined effect of interleukin-6 and D-dimer on the risk of serious non-AIDS conditions: data from 3 prospective cohorts. 20th Conference on Retroviruses and Opportunistic Infections; Atlanta, GA: CROI, LLC; 2013.

[6] Wilson EM, Singh A, Hullsiek KH, Gibson D, Henry WK, Lichtenstein K, et al Monocyte-activation phenotypes are associated with biomarkers of inflammation and coagulation in chronic HIV infection. The Journal of infectious diseases. 2014;210(9):1396–406. PubMed Central PMCID: PMCPMC4207864. doi: 10.1093/infdis/jiu275 24813472 10.1093/infdis/jiu275 PMC4207864

[7] Baker JV, Hullsiek KH, Singh A, Wilson E, Henry K, Lichtenstein K, et al Immunologic predictors of coronary artery calcium progression in a contemporary HIV cohort. Aids. 2014;28(6):831–40. PubMed Central PMCID: PMCPMC4199584. doi: 10.1097/QAD.0000000000000145 24370480 10.1097/QAD.0000000000000145 PMC4199584

[8] Vellozzi C, Brooks JT, Bush TJ, Conley LJ, Henry K, Carpenter CC, et al The study to understand the natural history of HIV and AIDS in the era of effective therapy (SUN Study). American journal of epidemiology. 2009;169(5):642–52. doi: 10.1093/aje/kwn361 19074775 10.1093/aje/kwn361

[9] Holick MF, Binkley NC, Bischoff-Ferrari HA, Gordon CM, Hanley DA, Heaney RP, et al Evaluation, treatment, and prevention of vitamin D deficiency: an Endocrine Society clinical practice guideline. The Journal of clinical endocrinology and metabolism. 2011;96(7):1911–30. doi: 10.1210/jc.2011-0385 21646368 10.1210/jc.2011-0385

[10] Dao CN, Patel P, Overton ET, Rhame F, Pals SL, Johnson C, et al Low vitamin D among HIV-infected adults: prevalence of and risk factors for low vitamin D Levels in a cohort of HIV-infected adults and comparison to prevalence among adults in the US general population. Clinical infectious diseases: an official publication of the Infectious Diseases Society of America. 2011;52(3):396–405. 21217186 10.1093/cid/ciq158

[11] Krishnan S, Wilson EM, Sheikh V, Rupert A, Mendoza D, Yang J, et al Evidence for innate immune system activation in HIV type 1-infected elite controllers. The Journal of infectious diseases. 2014;209(6):931–9. PubMed Central PMCID: PMC3935475. doi: 10.1093/infdis/jit581 24185941 10.1093/infdis/jit581 PMC3935475

[12] Westhorpe CL, Maisa A, Spelman T, Hoy JF, Dewar EM, Karapanagiotidis S, et al Associations between surface markers on blood monocytes and carotid atherosclerosis in HIV-positive individuals. Immunology and cell biology. 2014;92(2):133–8. doi: 10.1038/icb.2013.84 24296810 10.1038/icb.2013.84

[13] Fabre-Mersseman V, Tubiana R, Papagno L, Bayard C, Briceno O, Fastenackels S, et al Vitamin D supplementation is associated with reduced immune activation levels in HIV-1-infected patients on suppressive antiretroviral therapy. Aids. 2014;500:14–00582. 10.1097/QAD.0000000000000472 25493593

[14] Coussens AK, Martineau AR, Wilkinson RJ. Anti-Inflammatory and Antimicrobial Actions of Vitamin D in Combating TB/HIV. Scientifica. 2014;2014:903680 PubMed Central PMCID: PMCPMC4102066. doi: 10.1155/2014/903680 25101194 10.1155/2014/903680 PMC4102066

[15] Dobnig H, Pilz S, Scharnagl H, Renner W, Seelhorst U, Wellnitz B, et al Independent association of low serum 25-hydroxyvitamin d and 1,25-dihydroxyvitamin d levels with all-cause and cardiovascular mortality. Arch Intern Med. 2008;168(12):1340–9. doi: 10.1001/archinte.168.12.1340 18574092 10.1001/archinte.168.12.1340

[16] Pilz S, Dobnig H, Nijpels G, Heine RJ, Stehouwer CD, Snijder MB, et al Vitamin D and mortality in older men and women. Clin Endocrinol (Oxf). 2009;71(5):666–72. 19226272 10.1111/j.1365-2265.2009.03548.x

[17] Hewison M. Vitamin D and immune function: an overview. The Proceedings of the Nutrition Society. 2012;71(1):50–61. doi: 10.1017/S0029665111001650 21849106 10.1017/S0029665111001650

