# Supplementary figures and images for: Vitamin D deficiency is associated with IL-6 levels and monocyte activation in HIV-infected persons
Source: PLoS One. 2017 May 2;12(5):e0175517. doi: 10.1371/journal.pone.0175517 (PMC5413041; doi:10.1371/journal.pone.0175517)

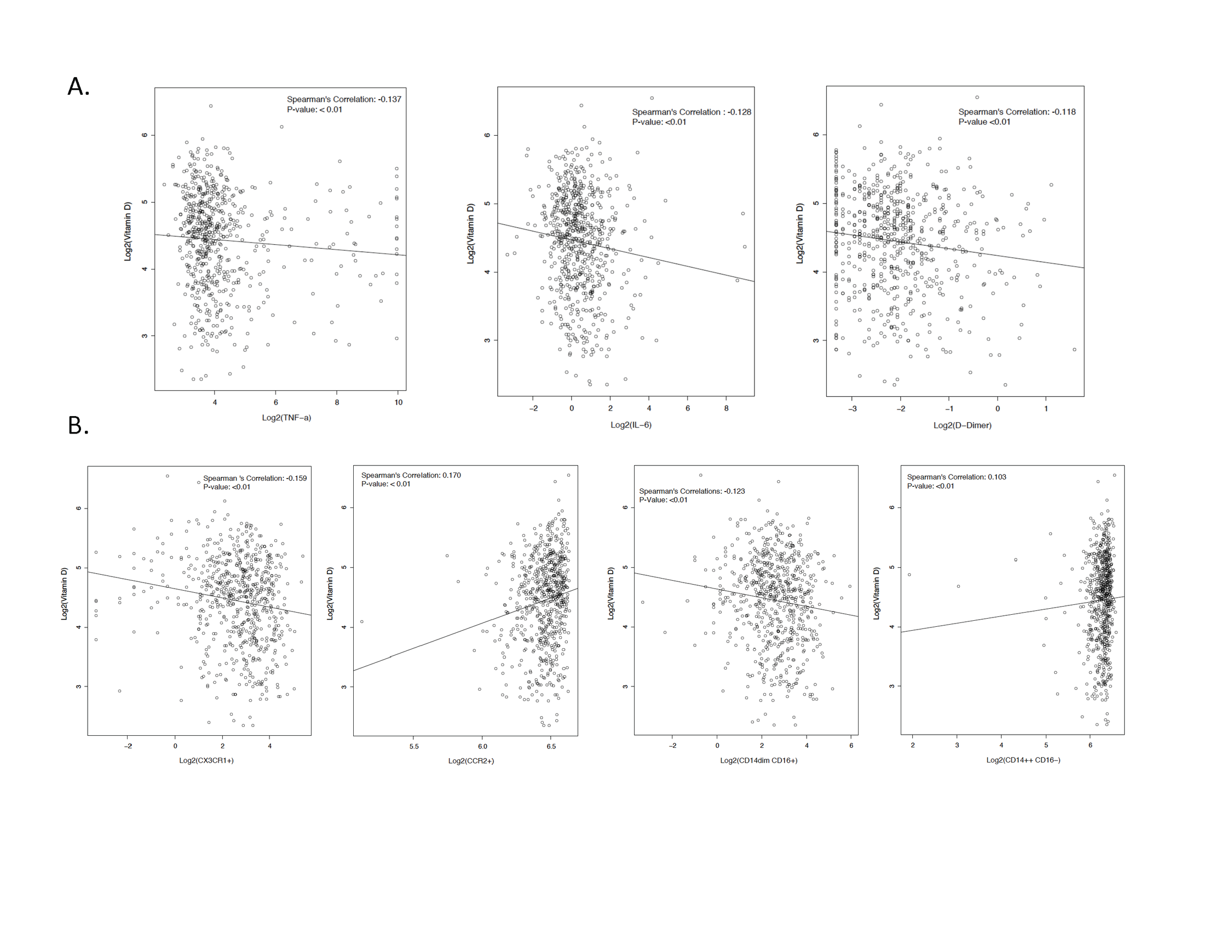

Supplement: S1 Fig — A demonstrates scatter plots of biomarkers by vitamin D and S1 Fig B demonstrates scatter plots of monocyte phenotypes by vitamin D. (TIFF) [file pone.0175517.s001.tiff]
